# FDG PET/CT to detect bone marrow involvement in the initial staging of patients with aggressive non-Hodgkin lymphoma: results from the prospective, multicenter PETAL and OPTIMAL>60 trials

**DOI:** 10.1007/s00259-021-05348-6

**Published:** 2021-04-29

**Authors:** Dominic Kaddu-Mulindwa, Bettina Altmann, Gerhard Held, Stephanie Angel, Stephan Stilgenbauer, Lorenz Thurner, Moritz Bewarder, Maren Schwier, Michael Pfreundschuh, Markus Löffler, Karin Menhart, Jirka Grosse, Marita Ziepert, Ken Herrmann, Ulrich Dührsen, Andreas Hüttmann, Francesco Barbato, Viola Poeschel, Dirk Hellwig

**Affiliations:** 1 Department of Hematology, Oncology, Clinical Immunology, Rheumatology, Medical School, University of Saarland, Kirrberger Str. 100, 66421 Homburg, Germany; 2 Institute for Medical Informatics, Statistics and Epidemiology, University Leipzig, Leipzig, Germany; 3 Department of Nuclear Medicine, University Hospital Regensburg, Regensburg, Germany; 4 Department of Nuclear Medicine, University Hospital Essen, University of Duisburg-Essen, Duisburg, Germany; 5 Department of Hematology, University Hospital Essen, University of Duisburg-Essen, Duisburg, Germany

**Keywords:** FDG PET/CT, Lymphoma, Aggressive B-cell lymphoma, Bone marrow biopsy, Diagnostic performance

## Abstract

**Purpose:**

Fluorine-18 fluorodeoxyglucose positron emission tomography combined with computed tomography (FDG PET/CT) is the standard for staging aggressive non-Hodgkin lymphoma (NHL). Limited data from prospective studies is available to determine whether initial staging by FDG PET/CT provides treatment-relevant information of bone marrow (BM) involvement (BMI) and thus could spare BM biopsy (BMB).

**Methods:**

Patients from PETAL (NCT00554164) and OPTIMAL>60 (NCT01478542) with aggressive B-cell NHL initially staged by FDG PET/CT and BMB were included in this pooled analysis. The reference standard to confirm BMI included a positive BMB and/or FDG PET/CT confirmed by targeted biopsy, complementary imaging (CT or magnetic resonance imaging), or concurrent disappearance of focal FDG-avid BM lesions with other lymphoma manifestations during immunochemotherapy.

**Results:**

Among 930 patients, BMI was detected by BMB in 85 (prevalence 9%) and by FDG PET/CT in 185 (20%) cases, for a total of 221 cases (24%). All 185 PET-positive cases were true positive, and 709 of 745 PET-negative cases were true negative. For BMB and FDG PET/CT, sensitivity was 38% (95% confidence interval [CI]: 32–45%) and 84% (CI: 78–88%), specificity 100% (CI: 99–100%) and 100% (CI: 99–100%), positive predictive value 100% (CI: 96–100%) and 100% (CI: 98–100%), and negative predictive value 84% (CI: 81–86%) and 95% (CI: 93–97%), respectively. In all of the 36 PET-negative cases with confirmed BMI patients had other adverse factors according to IPI that precluded a change of standard treatment. Thus, the BMB would not have influenced the patient management.

**Conclusion:**

In patients with aggressive B-cell NHL, routine BMB provides no critical staging information compared to FDG PET/CT and could therefore be omitted.

**Trial registration:**

NCT00554164 and NCT01478542

**Supplementary Information:**

The online version contains supplementary material available at 10.1007/s00259-021-05348-6.

## Background

Non-Hodgkin lymphoma (NHL) is responsible for about 6% of all cancer deaths in the western world [1]. Diffuse large B-cell lymphoma (DLBCL) is the most common subgroup accounting for 30–58% of lymphoma patients [2, 3]. Bone marrow involvement (BMI) may influence prognosis and treatment of aggressive B-cell NHL. The addition of the anti-CD20 antibody rituximab to CHOP polychemotherapy (doxorubicin, cyclophosphamide, vincristine, prednisone) has significantly improved the outcome of patients with DLBCL [4, 5]. The International Prognostic Index (IPI) can predict prognosis [6, 7]. An essential part of the IPI is the clinical stage, and BMI is classified as stage IV [8], indicating a less favorable prognosis. The recently published FLYER study demonstrated that the number of chemotherapy cycles can be reduced in patients younger than 60 years with low-risk IPI [9]. Taken together, the initial determination of the clinical stage including BMI remains crucial for treatment decisions and prognosis.

Study results show that, due to its higher sensitivity and specificity, fluorine-18 fluorodeoxyglucose positron emission tomography combined with computed tomography (FDG PET/CT) is superior to computed tomography (CT) alone in the detection of lymphoma manifestations [10]. It is therefore recommended for staging [11].

For decades, bone marrow biopsy (BMB) has been the standard for the evaluation of BMI in lymphoma. Evidence has been presented that BMI may also be detected by FDG PET/CT in patients with aggressive B-cell NHL. According to a recent meta-analysis, FDG PET/CT detects initial BMI with higher sensitivity than BMB, suggesting that BMB may be dispensable [12]. The meta-analysis included a total of 654 patients from 7 studies (2 prospective, 3 retrospective, 2 with unreported design) with up to 133 cases and a maximum of 55 patients in a prospective study [12]. In a pooled estimator, the sensitivity and specificity of FDG PET/CT for BMI were 88.7% (CI: 82.5–93.3%) and 99.8% (CI: 98.8–100%), respectively [12]. More recent prospective studies with concordant results addressed the performance of FDG PET/CT compared to BMB for staging and BMI in mixed populations of 68 patients with FDG-avid lymphoma including 16 DLBCLs [13] or 35 patients with aggressive NHL, respectively [14]. To the best of our knowledge, there is no large prospective study on the performance of FDG PET/CT in comparison to BMB for the detection of BMI in aggressive B-cell NHL.

Our aim was to analyze the diagnostic performance and relevance of FDG PET/CT for initial staging regarding BMI in aggressive B-cell NHL using data from two large prospective randomized multicenter trials of high methodological quality. We set out to answer the question whether noninvasive FDG PET/CT can diagnose BMI more reliably than invasive BMB does, rendering the latter dispensable.

## Methods

**Fig. 1:**
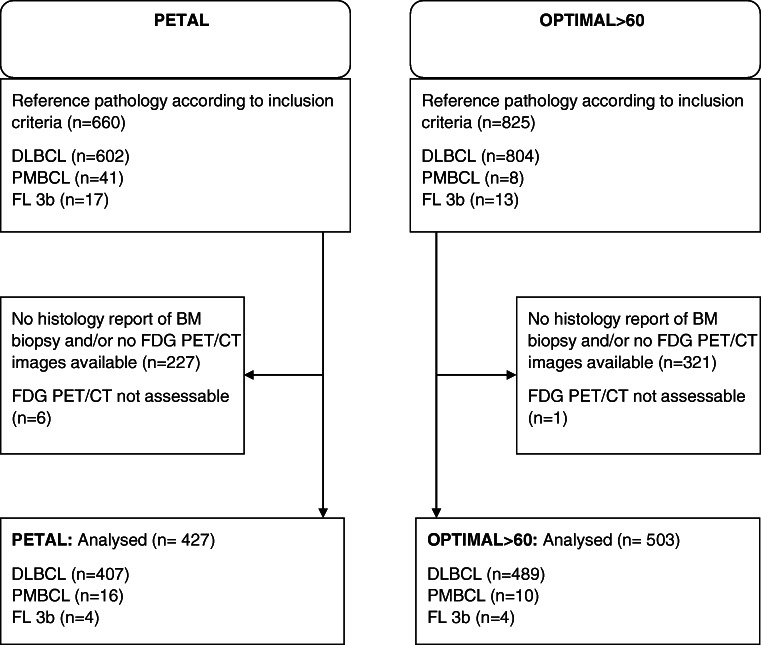
Consort diagram of patients from the PETAL and OPTIMAL>60 trials included in the bone marrow analysis

The present analysis was requested by the German Joint Federal Committee (Gemeinsamer Bundesauschuss, G-BA), the highest council of collective self-government of the German healthcare system under supervision of the German Federal Ministry of Health. The investigation used the data from two German prospective phase 3 trials: the PETAL study (NCT00554164) [15] and the OPTIMAL>60 study (NCT01478542). The first investigated whether FDG PET/CT can guide therapy in patients with aggressive NHL, whereas the second is an ongoing randomized trial including elderly patients with aggressive B-cell NHL with the purpose of improving outcome and reducing toxicity by using an optimized schedule of rituximab, substituting conventional by liposomal vincristine, and PET-guided reduction of therapy. The results presented here do not represent endpoints of the studies and have no influence on the endpoints or the conduct of the ongoing OPTIMAL>60 trial. According to the trial protocols, both a BMB and an FDG PET/CT were performed during initial staging. Patients were excluded from the analysis if a diagnosis of aggressive B-cell lymphoma could not be confirmed by reference pathology, if BMB or FDG PET/CT were not done, or if the BMB or imaging data were unavailable for central review. In order to ensure comparability of the patient cohorts, only patients with a biopsy-proven, centrally confirmed diagnosis of DLBCL, primary mediastinal B-cell lymphoma (PMBCL), or follicular lymphoma grade 3b (FL3b) were included (Fig. 1).

### Central reading of PET/CT

All FDG PET/CT images were centrally reviewed for BMI by an one expert from a panel of nuclear medicine physicians (DH, JG, KM, FB, KH) with board certifications for CT (DH, JG, KM) and MRI (DH) who were blinded to the results of BMB. PET/CT images were obtained as pseudonymized DICOM data sets from the study centers. PET/CT images were visualized as reported in the Supplemental Materials.

### PET criteria for BMI

The distribution pattern of FDG uptake in BM was rated as normal, diffusely increased, or unifocal/multifocal. PET positivity was assessed visually and required an FDG uptake level above that in normal liver. Diffuse uptake (“uniform increased FDG uptake throughout the bone marrow space” [16]), without focal uptake, was considered negative for BMI; otherwise, it was considered positive. Namely, diffuse uptake with concurrent focal uptake and areas of asymmetric diffuse uptake were considered involved. Foci attributable to physiologic conditions (e.g., rib fractures or sites of previous BMB) were considered negative.

### Study-related evaluation of FDG uptake in BM and of osseous involvement

For semiquantitative assessment in the present analysis, the intensity of FDG uptake in BM or in BM foci was rated by a 5-point scale in relation to mediastinal blood pool (MBP) and liver (1: no uptake, 2: less than or equal to MBP, 3: less than or equal to the liver, 4: moderately above the liver, 5: markedly above the liver) [10]. The anatomic localization of foci in the BM with increased FDG uptake was documented for further comparison. Osseous involvement was defined by an affection of the bone detected by CT as a lytic lesion or by MRI as a bone-invading lesion in cortical bone or in the cancellous bone (spongiosa) at the site of FDG uptake. If there was no anatomic imaging (full-dose CT, low-dose CT, or MRI) of adequate quality available for comparison in suspicious bone lesions, the presence of bone involvement was classified as “unknown” for these foci.

### Comparison of PET/CT and BMB findings

In a first step, discordant cases of BMB and blinded central PET/CT reading were identified. Discordant findings were documented and resolved after unblinding by re-evaluation in consensus with at least one further panel member and interdisciplinary discussion using findings of complementary imaging and/or subsequent PET/CT examinations, if available. In case of an FDG PET/CT result positive for BMI with negative BMB, all available additional imaging data (mainly CT or targeted magnetic resonance imaging, MRI) and/or FDG PET/CT image data sets from restaging or follow-up examinations were used to determine the final diagnosis of BMI.

### Definition of BM involvement

Based on the published criteria of Berthet and coworkers [17], a newly defined gold standard was used as reference standard for BMI. It included a positive BMB, a positive FDG PET/CT in discordant cases confirmed by targeted biopsy or complementary CT imaging (if available CT series from PET/CT were nondiagnostic, e.g., due to limitiations from low-dose CT protocols), or targeted MRI or concurrent disappearance of focal FDG PET-avid lesions in the BM together with other lymphoma manifestations after immunochemotherapy.

Identification of bone marrow infiltration was done using unilateral iliac crest biopsy. Marrow biopsy specimens were reported by a reference hematopathologist. The presence of lymphoma was based on standard immunohistochemistry, including antibodies to identify CD3-, CD79a-, and CD20-positive cells.

### Statistical analysis

To investigate whether the subgroups analyzed in this study were representative of the total trial populations, characteristics of included and excluded patients were compared using the chi^2^, Fisher exact, and Wilcoxon rank sum tests. Sensitivity, specificity, positive predictive value (PPV), and negative predictive value (NPV) were calculated with 95% confidence intervals (CI) to assess the diagnostic performance of FDG PET/CT and BMB in relation to the newly defined gold standard. For the comparison of sensitivities, the McNemar test was used [18]. Statistical evaluation was performed using SPSS Statistics 25 software (SPSS, Chicago, IL). The 95% Clopper-Pearson CIs were determined in Cytel Studio 8.0.

## Results

### Patient characteristics

From the PETAL and the OPTIMAL>60 trials, subgroups of 660 and 825 patients, respectively, with histopathologically confirmed aggressive B-cell NHL of the subtypes DLBCL, PMBCL, and FL3b, were eligible for this pooled analysis. In 433 and 504 cases, both PET/CT images and BMB reports were available and suitable for analysis in 427 and 503 patients, respectively (Fig. 1). Characteristics were similar in patients included and excluded from this analysis (Supplemental Table 1).

The demographic and clinical characteristics of the analysis population are shown in Table 1. Overall, both cohorts (PETAL and OPTIMAL>60) had similar characteristics, except for age, which was due to the inclusion criteria of the trials (the PETAL study included patients 18–80 years of age; the OPTIMAL>60 study only includes patients aged 61–80 years). The median age of all analyzed patients was 68 years, 56% were male, and 54% had an advanced stage. According to BMB, 36 of the 427 patients from PETAL (8%) and 49 of the 503 patients from OPTIMAL>60 (10%) had BMI; thus, BMB confirmed BMI in 85 of 930 patients in total (9%). According to initial FDG PET/CT, 88 of the 427 patients from the PETAL study (21%) and 97 of the 503 patients from the OPTIMAL>60 study (19%) had BMI, i.e., in total 185 of 930 patients (20%), as shown in Table 1. All 185 patients with BMI diagnosed by FDG PET/CT had an FDG uptake in the BM at least greater than the intensity of uptake in normal liver (Table 1), with unifocal, multifocal, or diffusely increased FDG uptake pattern in 44 (24%), 118 (64%), and 23 (12%) patients, respectively (Table 1). Of these 185 patients, 103 (56%) had concomitant osseous involvement at the site of the BM lesion; 70 (38%) had no osseous involvement; and in 12 cases (6%), correlative imaging of adequate quality was not available for central reading so that osseous involvement was judged “unknown.” In only 50% of the patients were the FDG-avid BM lesions located at the site commonly used for BMB (posterolateral iliac crest).

**Table 1:** Patients’ baseline characteristics

	PETAL *n* = 427	OPTIMAL>60 *n* = 503	Total *n* = 930
Male	235 (55%)	290 (58%)	525 (56%)
Female	192 (45%)	213 (42%)	405 (44%)
Age, median (range)	61* (18–80)	71 (61–80)	68 (18–80)
Age > 60 years	214* (50%)	503 (100%)	717* (77%)
LDH > UNL	247* (58%)	255 (51%)	502* (54%)
ECOG > 1	39* (9%)	23 (5%)	62* (7%)
Stage III/IV	239* (56%)	262 (52%)	501* (54%)
Extralymphatic inv. > 1	130* (31%)	151 (30%)	281* (30%)
IPI 0, 1	166* (39%)	140 (28%)	306* (33%)
IPI 2	98* (23%)	137 (27%)	235* (25%)
IPI 3	98* (23%)	138 (27%)	236* (25%)
IPI 4, 5	64* (15%)	88 (17%)	152* (16%)
Bone marrow involvement by BMB	36 (8%)	49 (10%)	85 (9%)
B-symptoms**	133 (31%)	106 (21%)	239 (26%)
Reference pathology			
DLBCL	407 (95%)	489 (97%)	896 (96%)
PMBCL	16 (4%)	4 (1%)	20 (2%)
Follicular lymphoma 3b	4 (1%)	10 (2%)	14 (2%)
Bone marrow involvement by FDG PET/CT	88 (21%)	97 (19%)	185 (20%)
Intensity of FDG uptake			
Uptake moderately > liver	4 (4%)	5 (5%)	9 (5%)
Uptake markedly increased > liver	84 (96%)	92 (95%)	176 (95%)
Type of lesion in FDG PET/CT			
Unifocal	26 (30%)	18 (19%)	44 (24%)
Multifocal	43 (49%)	75 (77%)	118 (64%)
Diffuse	19 (22%)	4 (4%)	23 (12%)
Skeletal involvement			
Osseous involvement	40 (46%)	63 (65%)	103 (56%)
No osseous involvement	38 (43%)	32 (33%)	70 (38%)
Unknown	10 (11%)	2 (2%)	12 (6%)
BM localization in FDG PET/CT			
Pelvis (dorsal) or sternum affected	42 (48%)	51 (53%)	93 (50%)
Pelvis (dorsal) and sternum unaffected	44 (50%)	46 (47%)	90 (49%)
Unknown	2 (2%)	0 (0%)	2 (1%)
Bone marrow involvement by reference standard	98 (23%)	123 (24%)	221 (24%)

Abbreviations: *BM*, bone marrow; *BMB*, bone marrow biopsy; *DLBCL*, diffuse large B-cell lymphoma; *ECOG*, Eastern Cooperative Oncology Group performance status; *FDG* PET/CT, fluorine-18 fluorodeoxyglucose positron emission tomography/computed tomography; *IPI*, International Prognostic Index; *LDH*, lactate dehydrogenase; *PMBCL*, primary mediastinal B-cell lymphoma; *ULN*, upper limit of normal

*One patient with missing values for single IPI factors and IPI score

**9 (PETAL *n* = 1/OPTIMAL>60 *n* = 8) unknown values

### Comparison of initial FDG PET/CT and BMB

**Fig. 2:**
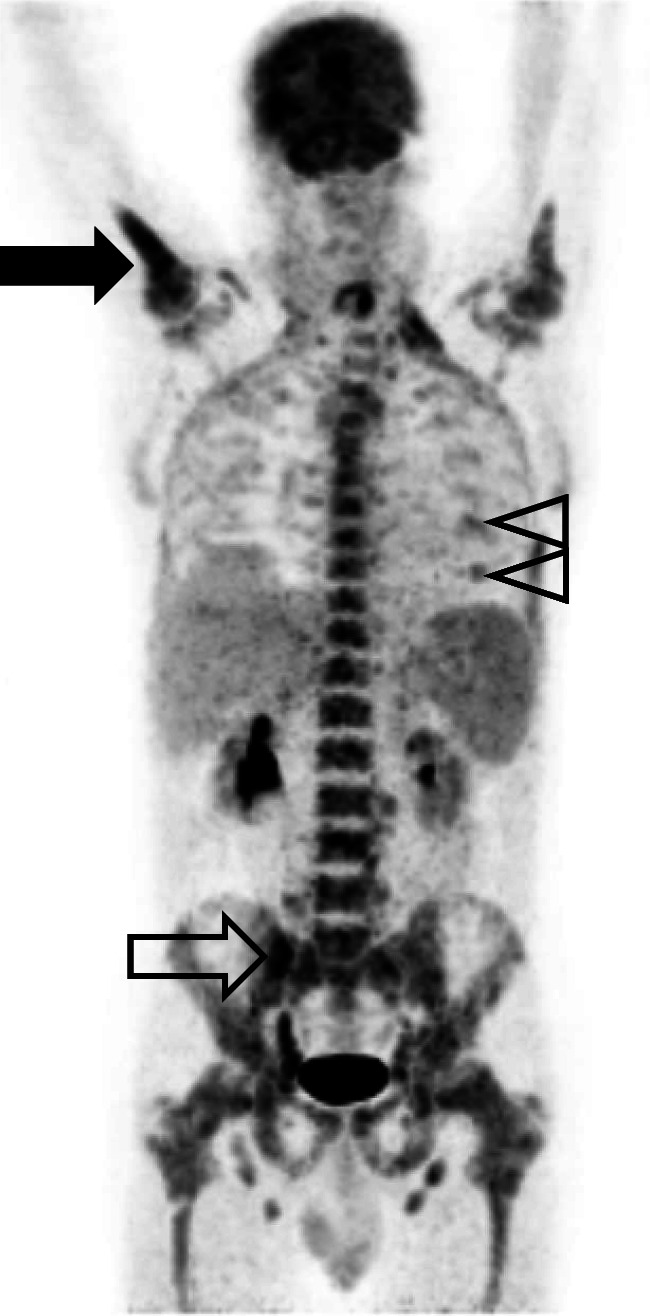
Baseline FDG PET (maximumintensity projection in frontal view) of a male patient with diffuse large B-cell lymphoma. FDG uptake in bone marrow space (with an intensity markedly above that in the liver) is diffusely increased without any focus in the bone marrow space apart from the biopsy site (right iliac crest, outlined arrow), but with asymmetric uptake (filled arrow) in right and left proximal humerus (maximum standardized uptake value 7.0 and 3.6, respectively) consistent with bone marrow involvement as confirmed by bone marrow biopsy. CT (not shown) revealed no osseous involvement in the proximal right humerus. Focal uptake in cartilage-bone junctions (outlined arrow-heads) as foci attributable to benign conditions

Of the 930 patients analyzed, 709 (76%) had a negative baseline FDG PET/CT and a negative BMB, whereas 49 (5%) had a positive FDG PET/CT and a positive BMB as demonstrated in Fig. 2. Thirty-six patients (4%) had a discordant result with FDG PET/CT negative for BMI but positive BMB. Among these 36 patients, 17 (47%, 3 in PETAL, 14 in OPTIMAL>60) had an indolent (discordant) NHL in the BMB, whereas the other 19 patients had an aggressive (concordant) B-cell lymphoma. One hundred thirty-six patients (15%) had a discordant result with a positive FDG PET/CT and a negative BMB (Table 2).

**Fig. 3:**
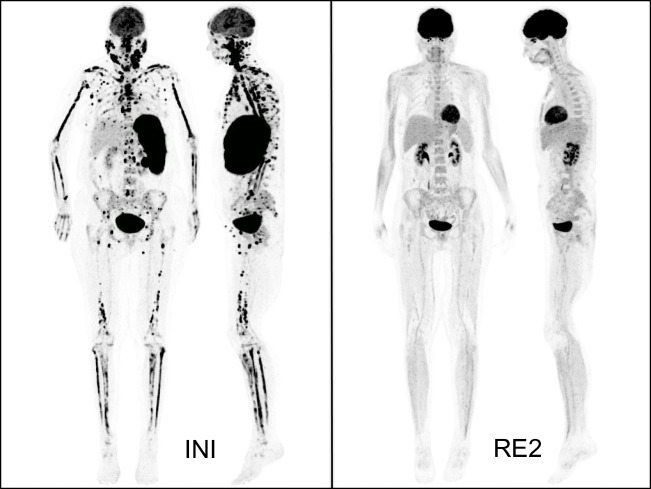
Female patient with diffuse large B-cell lymphoma and multifocal bone marrow involvement at baseline FDG PET/CT (INI), which was missed by bone marrow biopsy and remitted completely after 6 cycles of chemoimmunotherapy (RE2). Maximum-intensity projections in frontal and lateral view

**Table 2:** Comparison of the bone marrow biopsy results, the baseline FDG PET/CT results, and the newly defined gold standard regarding bone marrow involvement, respectively

		PETAL *n* = 427	OPTIMAL>60 *n* = 503	Total *n* = 930
BMB	FDG PET/CT			
Negative	Negative	329 (77%)	380 (76%)	709 (76%)
Negative	Positive	62 (15%)	74 (15%)	136 (15%)
Positive	Negative	10 (2%)	26 (5%)	36 (4%)
Positive	Positive	26 (6%)	23 (5%)	49 (5%)
BMB	Reference standard			
Negative	Negative	329 (77%)	380 (76%)	709 (76%)
Negative	Positive	62 (15%)	74 (15%)	136 (15%)
Positive	Negative	0 (0%)	0 (0%)	0 (0%)
Positive	Positive	36 (8%)	49 (10%)	85 (9%)
FDG PET/CT	Reference standard			
Negative	Negative	329 (77%)	380 (76%)	709 (76%)
Negative	Positive	10 (2%)	26 (5%)	36 (4%)
Positive	Negative	0 (0%)	0 (0%)	0 (0%)
Positive	Positive	88 (21%)	97 (19%)	185 (20%)

Abbreviations: *BMB*, bone marrow biopsy; *FDG PET/CT*, fluorine-18 fluorodeoxyglucose positron emission tomography/computed tomography

### Re-evaluation of discordant findings with imaging

Differences between BMB and FDG PET/CT were further reviewed by the expert panel. In the OPTIMAL>60 study, re-evaluation included further imaging (CT, MRI) and FDG PET/CT follow-up examinations. In 69 of the 74 cases with positive FDG PET/CT and negative BMB, FDG uptake completely or partially disappeared in the course of therapy, compatible with initial BMI. Figure 3 illustrates a typical case. The 5 remaining patients had no FDG PET/CT follow-up scans. They had unifocal (*n* = 2) or multifocal (*n* = 3) FDG uptake in the BM with involvement of the humerus, femur, and digits. According to CT criteria, these lesions were classified as BMI. Thus, all 74 patients with positive FDG PET/CT and negative BMB had convincing evidence of true BMI, which, however, remained undetected by BMB (Supplemental Table 2). The 62 discordant cases of the PETAL could not be further evalutated, because FDG PET/CT follow-up scans and/or additional imaging procedures were not available.

### Final diagnosis of BMI

The reference standard for BMI resulted in 709 cases that were negative, and 221 cases that were positive for BMI, with a prevalence of BMI of 221/930 = 24%.

### Diagnostic performance of BMB for the detection of BMI

Since 709 of the 845 BMB-negative cases were also negative according to the reference standard, the NPV of BMB was 84% (CI: 81–86%) (Table 3). The sensitivity of BMB was 38% (CI: 32–45%), since 85 of 221 cases diagnosed by the reference standard were also identified by BMB. All 85 BMB-positive cases were also positive by the reference standard, so the PPV was 100% (CI: 96–100%). The specificity was also 100% (CI: 99–100%) (Table 3).

**Table 3:** Diagnostic test parameters with 95% confidence intervals of the bone marrow biopsy and baseline FDG PET/CT results in comparison to the newly defined gold standard regarding bone marrow involvement, respectively

	PETAL *n* = 427		OPTIMAL>60 *n* = 503		Total *n* = 930	
BMB versus reference standard						
Sensitivity	36/98 (37%)	(27%; 47%)	49/123 (40%)	(31%; 49%)	85/221 (38%)	(32%; 45%)
Specificity	329/329 (100%)	(99%; 100%)	380/380 (100%)	(99%; 100%)	709/709 (100%)	(99%; 100%)
Positive predictive value	36/36 (100%)	(90%; 100%)	49/49 (100%)	(93%; 100%)	85/85 (100%)	(96%; 100%)
Negative predictive value	329/391 (84%)	(80%; 88%)	380/454 (84%)	(80%; 87%)	709/845 (84%)	(81%; 86%)
FDG PET/CT versus reference standard						
Sensitivity	88/98 (90%)	(82%; 95%)	97/123 (79%)	(71%; 86%)	185/221 (84%)	(78%; 88%)
Specificity	329/329 (100%)	(99%; 100%)	380/380 (100%)	(99%; 100%)	709/709 (100%)	(99%; 100%)
Positive predictive value	88/88 (100%)	(96%; 100%)	97/97 (100%)	(96%; 100%)	185/185 (100%)	(98%; 100%)
Negative predictive value	329/339 (97%)	(95%; 99%)	380/406 (94%)	(91%; 96%)	709/745 (95%)	(93%; 97%)

Abbreviations: *BMB*, bone marrow biopsy; *FDG PET/CT*, fluorine-18 fluorodeoxyglucose positron emission tomography/computed tomography

### Diagnostic performance of FDG PET/CT for the detection of BMI

As compared to the reference standard, 185 of 221 cases with BMI were detected by FDG PET/CT (Table 3), resulting in a sensitivity of 84% (CI: 78–88%). On the other hand, 709 of the 745 reference standard-negative cases were also found to be negative by FDG PET/CT, resulting in a NPV of 95% (CI: 93–97%). All 185 PET-positive cases were confirmed to represent BMI by central review, resulting in a PPV of 100% (CI: 98–100%). Of the 709 PET-negative findings, all were confirmed to be negative, so the specificity was 100% (CI: 99–100%).

The sensitivity of FDG PET/CT was significantly higher than that of BMB (84% versus 38%; *p* < 0.001).

### Effect of BMI detection on stage

Lactate dehydrogenase (LDH) levels were increased above the upper limit of normal in 26 of the 36 BMI cases that were missed by FDG PET/CT. From the remaining 10 patients, one had an ECOG performance status > 1 and 9 were in stage III/IV. Thus, every single patient whose BMI was missed by FDG PET/CT had another adverse characteristic according to IPI precluding eligibility for reduced chemotherapy [9].

On the other side, we observed that in 136 patients with an FDG PET/CT positive for BMI but negative BMB, a portion of 80% was already in stage III/IV according to CT-based Ann Arbor staging. Therefore, 27 patients (BMB negative but FDG PET/CT positive for BMI) would be upstaged (from stage I/II to stage III/IV) by FDG PET/CT.

## Discussion

The current analysis is derived from a comprehensive data set which originates from two large prospective randomized multicenter phase 3 trials. To our knowledge, with 930 analyzed patients, this is the largest cohort from prospective studies for the comparison of FDG PET/CT and BMB to detect BMI. In comparison, Alzahrani et al. analyzed the value of routine BMB in 530 patients with DLBCL showing no additional diagnostic or prognostic value of BMB over PET/CT alone. In contrast to our analysis, their study was retrospective which may have led to a selection bias [19]. Due to the size and inclusion criteria of our trials, the risk of a significant selection bias appeared small. Both the PETAL and the OPTIMAL>60 trials included patients with aggressive B-cell NHL irrespective of stage or IPI risk group.

Based on BMB, DLBCL involves the BM in up to 27% of cases [20–22]. The prognostic impact of BMI varies according to the degree and type (concordant versus discordant) of infiltration [22]. In our analysis, the prevalence of BMI detected by BMB was only 9% (85 of 930 patients), reflecting low sensitivity (in our analysis only 38%). Our observation is consistent with recently published data [23]. In 74 patients treated in the OPTIMAL>60 trial, BMB performed on the iliac crest did not detect BMI, mainly because the involvement was focal rather than general. Focal involvement has previously been demonstrated in studies comparing bilateral versus unilateral BMB [24]. Taking FDG PET/CT and BMB together, 221 of 930 patients showed BMI leading to a prevalence of 24% which is in line with the observed frequency in other studies [16, 17, 19, 25, 26]. In our analysis, the PPV for FDG PET/CT compared to the reference standard was 100% (CI: 98–100%). Therefore, BMB is dispensable if BMI is detected by FDG PET/CT. The NPV of FDG PET/CT measured against the newly defined gold standard was 95% (CI: 93–97%), significantly higher than the NPV of BMB which was only 84% (CI: 81–86%).

One may ask how many of the patients with diffuse FDG uptake in BM above that in the liver had BMI. We can analyze this most accurately by looking at the data from the OPTIMAL>60 subpopulation where 26 patients exhibited diffuse FDG uptake in BM above that in the liver. In four (1 with moderately and 3 with markedly increased uptake), the PET pattern of inhomogeneous or asymmetric FDG uptake into the BM was interpreted (and confirmed) as BMI. PET was interpreted as negative for BMI in 22 patients with diffuse FDG uptake in BM (19 moderately and 3 markedly above the liver) with 6 false-negative cases (all with FDG uptake in BM only moderately above that in the liver). Thus, 10 of 26 patients (38%) with diffuse uptake above the liver had BMI (3 of 6 (50%) with markedly increased BM uptake and 7 of 20 (35%) with moderate uptake). Considering all false-negative BM PET results in the OPTIMAL>60 subpopulation, the pattern of FDG uptake was regarded as normal in 20 patients (1 with uptake less or equal to mediastinal blood pool, 12 with uptake less or equal to the liver, and 7 cases with FDG uptake in BM only moderately above that in the liver), while in 6 patients, it was diffusely increased but with a uniform appearance (all only moderately above the level of the liver). In most, namely 14 of the 26 patients (54%) with PET false negative for BMI, BMB reports explicitly contained the information about the presence of follicular (*n* = 5) or low-grade lymphoma (*n* = 9) in the BM specimen. These types of NHL are known on the one hand to potentially transform into aggressive lymphoma and on the other hand to exhibit limited FDG avidity [27] as a reason why they can be missed by PET.

Our data suggests that BMB can be restricted to a minority of patients with negative BM findings on PET/CT where detection of BMI would result in upstaging and a change of therapy. As of now, this situation is only encountered in young patients (age ≤ 60 years) without IPI risk factors or bulky disease in whom reduced chemotherapy has been shown to be equally effective as standard treatment [9]. Currently, two trials are testing whether immunochemotherapy can also be reduced in elderly good-risk patients (OPTIMAL>60 NCT01478542, LNH 2009-1B NCT01285765). In other patients, the impact of reduced chemotherapy has not yet been evaluated, i.e., they would receive standard treatment irrespective of the presence or absence of BMI. Therefore, BMB could be omitted. In our analysis, 26 of the 36 cases with BMI detected only by BMB had an elevated LDH, 1 had an ECOG performance status > 1, and 9 were stage III/IV, demonstrating that, even without a BMB, none of these cases would have been eligible for reduced chemotherapy. A BMB may also be discussed in selected other cases, such as patients in whom prophylactic central nervous system (CNS)–directed therapy is considered on the basis of the CNS-IPI whose result is influenced by the presence or absence of BMI [28].

Importantly, BMB is an invasive procedure whereas FDG PET/CT is noninvasive. Since side effects of BMB or FDG PET/CT as pre-trial examinations were not recorded in our data sets, there was no information available about adverse events resulting from these procedures. But without doubt, a BMB is more painful than a PET/CT is, and it is associated with the risk of adverse events such as infection or bleeding [29].

Berthet et al. proposed criteria to confirm BMI in cases exclusively diagnosed by FDG PET/CT [17]. As compared to the previous definition, the criteria used here also included the information obtained from targeted CT imaging (not only MRI) which is more commonly available than MRI.

The newly defined “gold” standard may have limitations. The specificity of FDG PET/CT may be limited due to potential FDG uptake in benign bone lesions [30] even though additional full-dose CT or MRI might help to characterize such lesions. Nevertheless, PET/CT in our analysis had a specificity of 100% with no false-positive findings. Diagnostic performance is improved as shown by the clarification of discordant findings by complementary imaging. We cannot exclude false-negative findings when both BMB and FDG PET/CT fail to demonstrate BMI. Whole-body MRI might provide similar sensitivity as FDG PET/CT for BMI, but this has only been prospectively evaluated in a limited number of aggressive NHL patients [14].

The prognostic value of BMI as detected by FDG PET/CT would be of interest. However, outcome data from the OPTIMAL>60 trial are not yet available, because it is an ongoing study.

Our analysis has some limitations, as it includes individual patient data from trials with different inclusion criteria. Most important are the differences in patient age since OPTIMAL>60 includes only patients aged 61 years and above. However, even with different inclusion criteria, no significant differences were observed between the studies in the incidence of BMI or other findings underlining the strength of our analysis.

In summary, baseline FDG PET/CT more accurately detects BMI than BMB, with considerably higher sensitivity of 84% versus 38%. By using FDG PET/CT, BMB can be omitted in the vast majority of patients with aggressive NHL. Our results lend further support to clinical practice guidelines recommending replacement of BMB by FDG PET/CT in the initial staging of DLBCL [11, 31].

## Supplementary information

ESM 1 (DOCX 18 kb)
