# Supplementary material for: FDG PET/CT to detect bone marrow involvement in the initial staging of patients with aggressive non-Hodgkin lymphoma: results from the prospective, multicenter PETAL and OPTIMAL>60 trials
Source: Eur J Nucl Med Mol Imaging. 2021 Apr 29;48(11):3550–9. doi: 10.1007/s00259-021-05348-6 (PMC8440256; doi:10.1007/s00259-021-05348-6)
Supplement: Supplementary file 1 — (DOCX 18 kb) [file 259_2021_5348_MOESM1_ESM.docx]

**Supplemental Table 1:**

**Clinical characteristics at initial staging for excluded and included patients of the PETAL and the OPTIMAL>60 cohort.**

|  | **PETAL** | | | | | **OPTIMAL>60** | | | | |
| --- | --- | --- | --- | --- | --- | --- | --- | --- | --- | --- |
|  | **Excluded**  **(n=233)** | | **Included**  **(n=427)** | | **p-value** | **Excluded**  **(n=322)** | | **Included**  **(n=503)** | | **p-value** |
| Male  Female | 135  98 | (58%)  (42%) | 235  192 | (55%)  (45%) | 0.472 | 175  147 | (54%)  (46%) | 290  213 | (58%)  (42%) | 0.350 |
| Age, median (range) | 58 | (18,80) | 61* | (18,80) | 0.051 | 71 | (61,80) | 71 | (61,80) | 0.904 |
| Age > 60 years | 103 | (44%) | 214* | (50%) | 0.139 | 322 | (100%) | 503 | (100%) | -- |
| LDH > UNL | 128 | (55%) | 247* | (58%) | 0.450 | 192 | (60%) | 255 | (51%) | 0.012 |
| ECOG > 1 | 23 | (10%) | 39* | (9%) | 0.763 | 21 | (7%) | 23 | (5%) | 0.224 |
| Stage III / IV | 140 | (60%) | 239* | (56%) | 0.323 | 187 | (58%) | 262 | (52%) | 0.092 |
| Extralymphatic inv. > 1 | 70 | (30%) | 130* | (31%) | 0.899 | 97 | (30%) | 151 | (30%) | 0.975 |
| IPI 0, 1 | 91 | (39%) | 166* | (39%) | 0.163 | 74 | (23%) | 140 | (28%) | 0.185 |
| IPI 2 | 68 | (29%) | 98* | (23%) |  | 80 | (25%) | 137 | (27%) |  |
| IPI 3 | 39 | (17%) | 98* | (23%) |  | 97 | (30%) | 138 | (27%) |  |
| IPI 4, 5 | 35 | (15%) | 64* | (15%) |  | 71 | (22%) | 88 | (17%) |  |
| Bone marrow involvement by BMB | 10 | (4%) | 36 | (8%) | 0.046 | 23 | (7%) | 49 | (10%) | 0.197 |
| B-symptoms | 69 | (30%) | 133** | (31%) | 0.669 | 97*** | (31%) | 106*** | (21%) | 0.003 |
| Reference pathology: |  |  |  |  |  |  |  |  |  |  |
| DLBCL | 195 | (84%) | 407 | (95%) | <0.001 | 315 | (98%) | 489 | (97%) | 0.489 |
| PMBCL | 25 | (11%) | 16 | (4%) |  | 4 | (1%) | 4 | (1%) |  |
| Follicular lymphoma 3b | 13 | (6%) | 4 | (1%) |  | 3 | (1%) | 10 | (2%) |  |
|  |  |  |  |  |  |  |  |  |  |  |

Abbreviations: BMB, bone marrow biopsy; DLBCL, diffuse large B-cell lymphoma; ECOG, Eastern Cooperative Oncology Group performance status; IPI, International Prognostic Index; LDH, lactate dehydrogenase; PMBCL, primary mediastinal B-cell lymphoma; ULN, upper limit of normal

* one patient with missing values for single IPI factors and IPI score

** one unknown value

*** 12 (excluded n=4/included n=8) unknown values

**Supplemental Table 2:**

**Validation of discordant cases (n=74) of the OPTIMAL>60 trial including complimentary imaging procedures and follow-up examinations.**

| **Further imaging** | **BM involvement verified** |
| --- | --- |
| Staging: CT part of FDG PET/CT | 1 |
| Staging: CT part of FDG PET/CT and  Staging: CT | 4 |
| Follow-up: FDG PET/CT | 39 |
| Staging: CT part of FDG PET/CT and  Follow-up: FDG PET/CT | 2 |
| Staging: CT and  Follow-up: FDG PET/CT | 1 |
| Staging: CT part of FDG PET/CT,  Staging: CT, and  Follow-up: FDG PET/CT | 22 |
| Staging: MRI and  Follow-up: FDG PET/CT | 2 |
| Staging: CT,  Staging: MRI, and  Follow-up: FDG PET/CT | 2 |
| Staging: CT part of FDG PET/CT,  Staging: CT,  Staging: MRI, and  Follow-up: FDG PET/CT | 1 |

Abbreviations: CT, computed tomography; FDG PET/CT, fluorine-18 fluorodeoxyglucose positron emission tomography/computed tomography; MRI, magnetic resonance imaging

# Supplemental Material

## Methods

Visualisation of PET/CT images

Osirix MD v.7.0.2 (Pixmeo Sarl, Bernex, Switzerland) was used for image data management and for reading PET/CT images. For PET reading, the images were presented with fixed lower and upper levels of 0 and 6 SUV (standardized uptake value), respectively. Attenuation corrected PET images were used for visual interpretation. Transverse images and maximum intensity projections (MIPs) of PET images were displayed using a linear inverse grey scale (no uptake: white, highest uptake: black, preset ‘B/W Inverse’ in Osirix MD). If SUV scaling was not possible (mostly due to missing information about the patient’s weight at the time of PET imaging) image scaling was performed manually by choosing the upper limit of the color lookup table such that the FDG uptake in liver corresponded to a medium grey color.

For CT reading, preferentially the series reconstructed with a bone kernel with slice spacing of 3 mm was used, otherwise a series reconstructed with a soft tissue kernel with a slice spacing between 1 and 5 mm was accepted for reading. CT images were displayed in a bone window (window level (WL) 300 Houndsfield units, HU, window width 1500 HU). Image fusion of PET and CT was done by a linear overlay using the color lookup table ‘NIH’ in Osirix MD. For the interpretation of extraosseous FDG accumulations, CT images were displayed using soft tissue windowing (WL 40 HU, WW 350 HU), preferentially from a series reconstructed with a soft tissue kernel. Orthogonal multiplanar reconstructions (MPRs) of fused PET and CT images were used for localization of suspected lesions. Reangulated MPRs were used for suspected BM lesions in long bones
